# *In Vitro* Folliculogenesis in Mammalian Models: A Computational Biology Study

**DOI:** 10.3389/fmolb.2021.737912

**Published:** 2021-11-09

**Authors:** Nicola Bernabò, Chiara Di Berardino, Giulia Capacchietti, Alessia Peserico, Giorgia Buoncuore, Umberto Tosi, Martina Crociati, Maurizio Monaci, Barbara Barboni

**Affiliations:** ^1^ Unit of Basic and Applied Biosciences, University of Teramo, Teramo, Italy; ^2^ National Research Council, Institute of Biochemistry and Cell Biology, Rome, Italy; ^3^ Department of Veterinary Medicine, University of Perugia, Perugia, Italy; ^4^ Centre for Perinatal and Reproductive Medicine, University of Perugia, Perugia, Italy

**Keywords:** ovarian folliculogenesis, computational biology, *In vitro* folliculogenesis network, hub molecules, bottleneck molecules

## Abstract

*In vitro* folliculogenesis (*iv*F) has been proposed as an emerging technology to support follicle growth and oocyte development. It holds a great deal of attraction from preserving human fertility to improving animal reproductive biotechnology. Despite the mice model, where live offspring have been achieved,in medium-sized mammals, *iv*F has not been validated yet. Thus, the employment of a network theory approach has been proposed for interpreting the large amount of *iv*F information collected to date in different mammalian models in order to identify the controllers of the *in vitro* system. The WoS-derived data generated a scale-free network, easily navigable including 641 nodes and 2089 links. A limited number of controllers (7.2%) are responsible for network robustness by preserving it against random damage. The network nodes were stratified in a coherent biological manner on three layers: the input was composed of systemic hormones and somatic-oocyte paracrine factors; the intermediate one recognized mainly key signaling molecules such as PI3K, KL, JAK-STAT, SMAD4, and cAMP; and the output layer molecules were related to functional *iv*F endpoints such as the FSH receptor and steroidogenesis. Notably, the phenotypes of knock-out mice previously developed for hub.BN indirectly corroborate their biological relevance in early folliculogenesis. Finally, taking advantage of the STRING analysis approach, further controllers belonging to the metabolic axis backbone were identified, such as mTOR/FOXO, FOXO3/SIRT1, and VEGF, which have been poorly considered in *iv*F to date. Overall, this *in silico* study identifies new metabolic sensor molecules controlling *iv*F serving as a basis for designing innovative diagnostic and treatment methods to preserve female fertility.

## Introduction

Assisted reproductive technologies (ARTs) represent a consolidated clinical practice, which have resulted in several million births since 1978 (Adamson et al., 2018) (International Federation of Fertility Societies’ Surveillance, 2019). However, the current ART protocols allow to use only a limited number of oocytes derived from antral follicles, while the large ovarian reserve, represented by the pool of early-stage follicles, remains a genetic patrimony that can be preserved by cryopreservation procedures but not managed (Martinez et al., 2017). In this context, the development of protocols aiming to obtain and fertilize mature oocytes from immature follicles grown outside the body could represent a useful strategy to recover the largest pool of ovarian gametes by promoting their *in vitro* growth and differentiation.

This follicle rescue approach could be applied as a possible future clinical strategy to preserve the ovarian reserve, as in the case of adult and prepubertal patients with cancer, where ovarian transplantation may expose to the risk of reintroducing malignant cells (Herta et al., 2018) (De Vos et al., 2014). Besides, it would benefit the veterinary medicine, providing a way to improve reproductive performance of species of zootechnical interest as well as to conserve the genetic inheritance of endangered animals.

Since the first attempt in the field of *in vitro* folliculogenesis (*iv*F) in 1996 (Eppig, 1996) (O’Brien et al., 2003), many efforts are made to set up new culture systems able to support *in vitro* growth of early-stage follicles toward competent oocytes (Laronda et al., 2017) (Xiao et al., 2015) (Xiao et al., 2017) (McLaughlin et al., 2018). The use of animal models to recapitulate *iv*F steps, driving to the production of fertilizable oocytes, has proven to be decisive for providing a knowledge basis and for developing validated methods with high translational potential for humans. Based on similarities in physiology and anatomy of the ovaries, folliculogenesis timing, and the follicle size (Barboni et al., 2011) (Bähr and Wolf, 2012) (Telfer and Zelinski, 2013), medium-sized mono-ovulatory mammals are commonly accepted as a translational model, and they are increasingly considered as being very relevant for human preimplantation reproductive research.

However, apart from the murine model where *in vitro* production of fertilizable oocytes has reached high levels of efficiency in terms of embryo development (Xu et al., 2006) (Hornick et al., 2013) (Hikabe et al., 2016), in medium-sized mammals, *iv*F remains still experimental. Indeed, a very low number of embryos produced from *in vitro* grown preantral follicles were reported in these models (de Figueiredo et al., 2018) even if several groups are working on bovine (Gupta et al., 2008) (Gupta and Nandi, 2012) (Antonino et al., 2019), porcine (Wu et al., 2001), caprine (Magalhães et al., 2011), ovine (Barboni et al., 2011) (Luz et al., 2012) (Arunakumari et al., 2010), and non-human primates (Xu et al., 2009) (Xu et al., 2011).

The difficulty to recapitulate *in vitro* the process of folliculogenesis in non-rodent animal models appeared to be related to the longer period required for follicle/oocyte growth, the greater dimension of antral follicles with competent oocytes, and the difficulty to mimic the environmentally favorable conditions to guarantee a synergic oocyte and somatic compartment development by preserving the tissue architecture (Rossetto et al., 2016).

Considering the large amount of data collected *in vitro* on the molecular mechanisms involved in the folliculogenesis among different species and the advances in *in vitro* follicle culture models, involving *in vitro* 2D and 3D culture approaches (Jones and Shikanov, 2019), the adoption of mathematical models might represent a valuable tool to organize the evidence collected to date by offering predictive models.

This study supports the use of a computational method based on network theory to identify the molecular events and the main factors sustaining *iv*F steps in mammals and to discover new molecular players in the *iv*F process to be targeted and/or exploited for therapeutic purposes, improving female fertility.

## Materials and Methods

### Data Collection: Web of Science-Mammals-Made *iv*F Database (WoS_MM*iv*F)

Scientific literature published in the peer-reviewed international indexes such as the Advanced Search of Web of Science (v.5.35) “Core collection” archive (https://apps.webofknowledge.com/WOS_AdvancedSearch) of the past 30 years was considered (Bernabò et al., 2010) (Bernabò et al., 2014) using the following key words: “*in vitro* culture”, “Follicle culture”, “*in vitro* folliculogenesis”, “Oocyte”, and “Ovary”. “AND” and “NOT” were used as Boolean operators, and “TS” was used as a field tag.

Each list obtained from manual data mining was matched to create a unique database “WoS_MMivF”, including exclusively mammalian-related manuscripts, which accounts totally for 1,111 papers. The quality control of manually collected data was carried out according to *Bernabò et al.*, (Bernabò et al., 2016).

The final WoS_MM*iv*F database contains 513 selected *iv*F-related manuscripts classified as original primary research articles (444/513; 87%) and reviews (69/;513; 13%), according to *Taraschi et al.* (Taraschi et al., 2020). The WoS_MM*iv*F database was enriched in Microsoft Excel 365 with the following fields (Supplementary File 1):

a) Source molecule: The molecule working as the source of interaction.
b) Interaction: The interaction the molecules carry out.
c) Target molecule: Molecules or molecular events that are the target of interaction.
d) Species: Different species of mammals in which molecular interactions occur.
e) Reference: PubMed IDentifiers (PMID).

Additional details related to the database set up can be found below:

The freely available and diffusible molecules such as H_2_O, CO_2,_ Pi, and H^+^O_2_ were mainly omitted. In case the target where a single molecular determinant of the phenomenon is unknown as a target, the related ovarian function was indicated (i.e., “preantral follicle growth” and “follicle activation”).

### *iv*F Network Creation, Visualization, and Analysis

The data, extracted from the database, were used to build the *iv*F network using the Cytoscape 3.6.0 software (http://www.cytoscape.org) (Paul et al., 1971). The network was analyzed with the specific plug-in Network Analyzer by computing the topological parameters described in Supplementary Data Sheet S2. The hubs, defined as hyperconnected nods, were identified as previously described (Bernabò et al., 2015b) (Bernabò et al., 2015a) by using the following equation: y> μ+ σ ,

where

γ = number of links per node (connectivity).

μ = mean node degree

σ = node degree standard deviation.

#### Closeness Centrality

Closeness centrality is a measure of how fast information spreads from one node to another reachable node (https://med.bioinf.mpi-inf.mpg.de/netanalyzer/help/2.7/index.html#refNewman2003).

This parameter is defined as the reciprocal of the average shortest path length and is computed as follows: Cc(n) = 1 / avg(L(n,m).

Here, L(n,m) is the length of the shortest path between two nodes n and m. The closeness centrality of each node is a number between 0 and 1. Network Analyzer computes the closeness centrality of all nodes and plots it against the number of neighbors. The closeness centrality of isolated nodes is equal to 0.

### Betweenness Centrality

***C***_***b***_ ***(n)*** of a node *n* is computed as follows: *C*_*b*_(*n*) = ∑_*s≠n≠t*_ (*σ*_*st*_ (*n*) / *σ*_*st*_), where *s* and *t* are nodes in the network different from *n*, *σ*_*st*_ denotes the number of shortest paths from *s* to *t*, and *σ*_*st*_ (*n*) is the number of shortest paths from *s* to *t* that *n* lies on. The betweenness centrality is computed only for networks that do not contain multiple edges. The betweenness value for each node *n* is normalized by dividing by the number of node pairs excluding *n*: (*N*-1) (*N*-2)*/2*, where *N* is the total number of nodes in the connected component that n belongs to. Thus, the betweenness centrality of each node is a number between 0 and 1 (https://med.bioinf.mpi-inf.mpg.de/netanalyzer/help/2.7/index.html#nodeBetween).

### Identification of Bottlenecks (CytoHubba)

Bottlenecks (BN) were identified as follows: let Ts be a shortest path tree rooted at node s, BN(v) = Σs∈V ps(v). In detail, e ps(v) = 1 if more than |V(Ts)|/ 4 paths from node s to other nodes in Ts meet at the vertex v; otherwise ps(v) = 0 (Chin et al., 2014) (Taraschi et al., 2020) (Ordinelli et al., 2018).

### In/Out Degree Ratio

In/out degree ratio (DR_IO_) was computed as follows:

$$DR_{io}=\frac{\gamma \;IN}{\gamma \;OUT}*100$$

It expresses the ratio between the number of links in the input and in the output for each node; consequently, it has been used to layer the nodes in input, processing, and output *strata* of the network.

### Network Topology Transition

To assess the relevance of network controllers in the maintenance of network stability, the removal of the most connected nodes on the network topology (targeted attack theory) was performed by means of two cycles of attacks, removing 2.5% of hubs each time. At the end of each attack, the network topology was examined.

### Enrichment Analysis

A Search Tool for the Retrieval of Interacting Genes/Proteins (STRING, http://string-db.org/newstring_cgi/show_input_page.pl?UserId=eNOo92_OQ_LS&sessionId=Cfz4mDP5ayne) (Szklarczyk et al., 2015) was used in order to enrich the database by including known and predicted protein interactions. They could be either direct (physical) or indirect (functional) associations and are derived from different sources: genomic context, high-throughput experiments, conserved coexpression, and previous knowledge. A new network was obtained (STRING_MM*iv*F) by adopting a medium confidence score (0.400). For the enrichment procedures, the false discovery rate (FDR) value was set to be <0.05, and 4 cycles of enrichment were performed.

### Gene Ontology

Gene Ontology (GO; https://www.geneontology.org) was carried out to identify the main functions, processes, and cellular compartments of the hub.BN and interactors according to their GO terms. The FRD value was set for *p* < 0.05.

## Results

The *iv*f process is described by a scale-free, non-clustered, and non-hierarchical system network.

Up to half of the links within the network are referred to rodents (39%) and ruminants (ovine 17.2, caprine 3.9, and bovine 13.7%), whereas less than 9% comes from humans (Table 1).

**TABLE 1:** WoS_MMivF database incidence of different mammal models.

Mammal models	Interactions (%)
Rodent	39.17
Human	8.76
Non-human primate	1.26
Porcine	7.14
Caprine	3.9
Ovine	17.2
Bovine	13.7
Canine	0.16
Feline	1.7
Leporid	0.2
Mammal*	6.85

The percentage of article related to each mammal model was calculated through the interaction count of the database WoS_MMi*v*F made using the manuscripts regarding the *iv*F protocols. *Asterisk refers to papers that did not discriminate among mammalian models.

Based on data collected in the WoS_MM*iv*F database, it was possible to generate a biological network whose topological parameters were computed as reported in Supplementary File 3.

The network displayed a scale-free topology, according to the Barabási-Albert (BA) model. The statistical analysis of its topology (Table 2) demonstrated that it recognizes 641 nodes and 2089 links, with a very low clustering coefficient (0.076).

**TABLE 2:** Topological parameter of biological networks made from the WoS_MMivF database.

Parameters	Value
Number of nodes	641
Number of links	2086
Number of connected components	1
Clustering coefficient	0.076
Char. path length	5.656
Avg. number of neighbors	5.042
In degree	—
Γ	−1.072
R	0.892
R^2^	0.701
Out degree	—
Γ	−1.252
R	0.995
R^2^	0.844

Table showing the results of topological analyses of the network obtained from the WOS_MM*iv*F database.

The main controllers of the network were identified: hub, bottleneck, and hub-bottleneck nodes.

The *iv*F network recognized as hyperconnected nodes (hubs) 5.9% of nodes (38 out of a total of 641) (Supplementary File 4), which were ranked based on node degree (Supplementary File 4). They include activating systemic hormones and ovarian activating factors (8 and 11 out of 38, respectively; overall 50% of hubs) more than transduction terminal events (4, 10, and 5%).

A Kernel density estimation (KDE) based on their clustering coefficient was then performed (see Supplementary File 4 for clustering coefficient value) in order to explore their distribution, showing that no node subpopulation is present (as reported in Supplementary File 5).

Furthermore, to assess the hub role in the *iv*F network stability control, a computational experiment was performed, carrying out subsequent cycles of hub removal (Supplementary File 6). The network topology was deeply affected by hub removal and collapsed upon two cycles of network attack. As comparison, the removal of the same number of nodes randomly identified [=random.between(min;max)] did not have detectable effects on network stability (*data not shown*).

In addition, seeking for controllers of the information flow within the network, 38 bottleneck nodes were identified (BN: Supplementary File 4) (Ordinelli et al., 2018) (Taraschi et al., 2020).

Finally, by intersecting the two subsets of nodes categorized as hubs and BN, 30 main nodes were identified and named hub.BN (Supplementary File 7).

### Role of hub.BN Nodes in the Scale-free *iv*f Network

More in detail, the hub.BN enclosed 10 functional events and 20 molecules.

Among the events, four stages of follicle development (primordial follicle activation, primordial to primary follicle transition, preantral follicle growth, and preantral to antral follicle transition), three key outcomes of the early stage of follicle development (antrum differentiation, steroidogenesis, and meiotic competence), and three cell functions controlling tissue homeostasis (cell survival, proliferation, and apoptosis) were identified.

**FIGURE 1:**
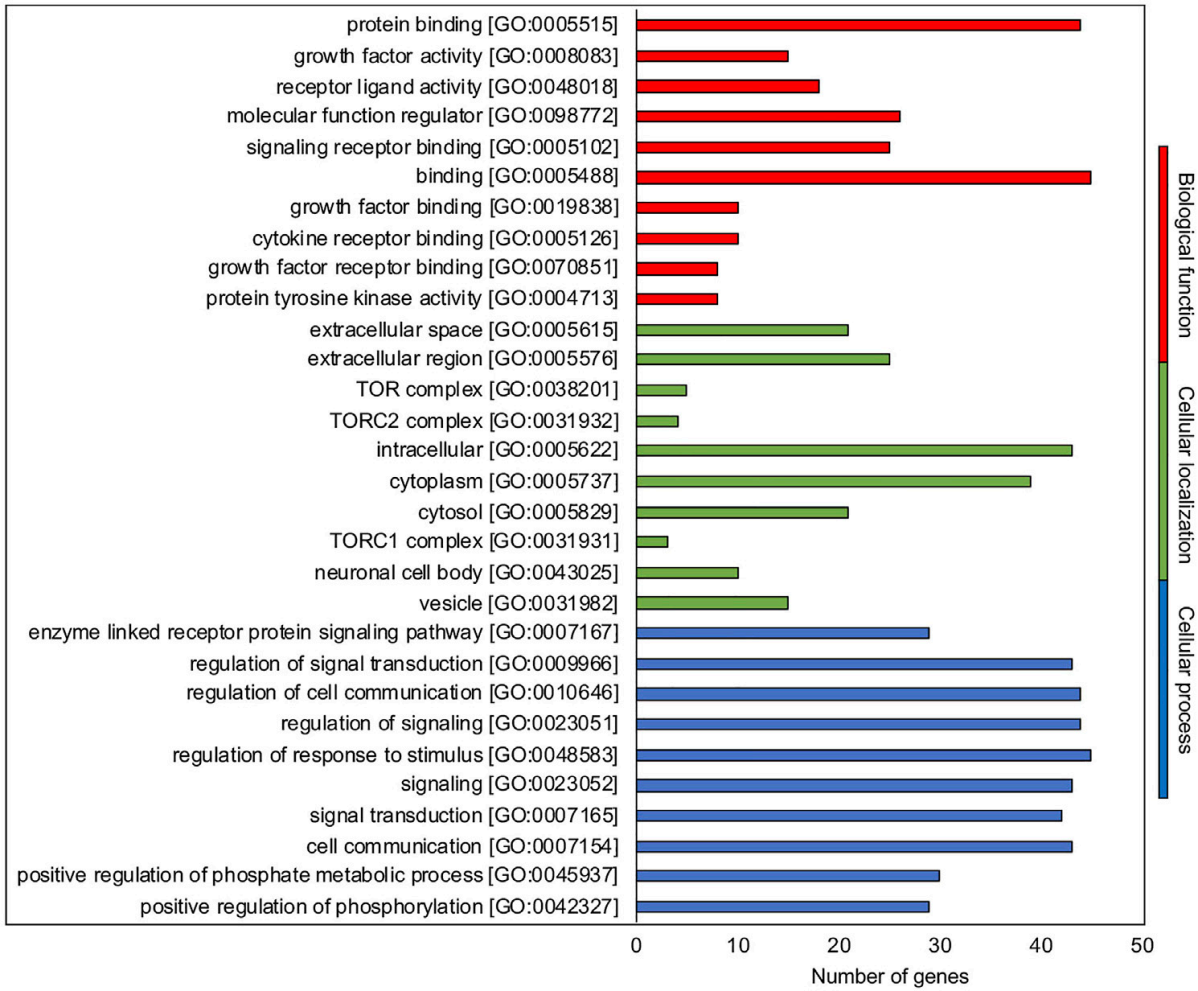
GO enrichment analysis. Representative scheme of the top 10 most abundant GO terms identified for the hub.BN molecules in the three GO categories: biological function (red), cellular localization (green), and biological processes (blue). The *x*-axis indicates the hub.BN molecules in a specific category, while the *y*-axis indicates different GO terms.

The 20 molecules belonging to hub.BN summarized in Supplementary File 7 based on their role in the ovarian folliculogenesis (hormone or paracrine/autocrine factors and driven follicular events) were classified for their biological function, process, and cellular localization (Figure 1) by using Gene Ontology (GO) (http://geneontology.org/) and setting the selection on the FDR value <0.05 (Supplementary File 8). The top 10 most abundant categories in terms of GO for hub.BN recognized hormone/paracrine binding (5 out of 10) and activities (4 out of 10) as key biological functions addressed to regulate the key cellular outcome processes of signaling (4 out of top 10 GO biological processes category) and cell to cell communication (1 out of top 10) inside the reproductive systems (4 out of 10). The main extracellular (3 out of 10) and secretory vesicle (3 out of 10) localization of hub.BN molecule actions was also highly consistent with the component GO category results.

Interestingly, knock-out mouse model data retrieved from the Mouse Genome International database (MGI, http://www.informatics.jax.org/) and WoS database (https://apps.webofknowledge.com) for the identified hub.BN genes showed altered phenotypes in the reproductive system (Supplementary File 9), supporting their key role as controllers of the network.

**FIGURE 2:**
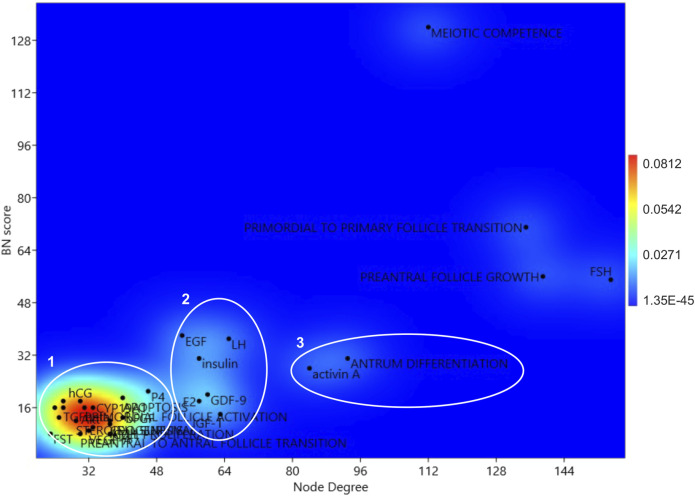
KDE 2D analysis of the network hubs. Centrality parameters of BN and node degree parameters of the hub.BN were considered for the 2D KDE analysis.

A 2D KDE analysis was then carried out to identify eventual subpopulation in the hub.BN population based on node degree and BN scores. Four isolated nodes and three subpopulations were identified (Figure 2). Four isolated hub.BN were characterized by higher values of node degree and BN score. Among them, it was possible to distinguish the main target of oogenesis related to the early stage of follicle development (acquisition of oocyte meiotic competence) and two follicular stages reproducible *in vitro* (primordial to primary follicle transition and preantral follicle growth). In addition, the 2D shape KDE computed analysis was assigned to this main subpopulation of hub.BN and also to the pituitary hormone FSH.

Subpopulations 3 and 2 recognized seven key endocrine/paracrine controllers of folliculogenesis (activin A and LH, GDF9, EGF, E2, Insulin, and IGF1) as well as the event of follicle specialization that occurs *in vitro* by applying the current protocols (antrum differentiation). The remaining hub.BN belongs to subpopulation 1.

**FIGURE 3:**
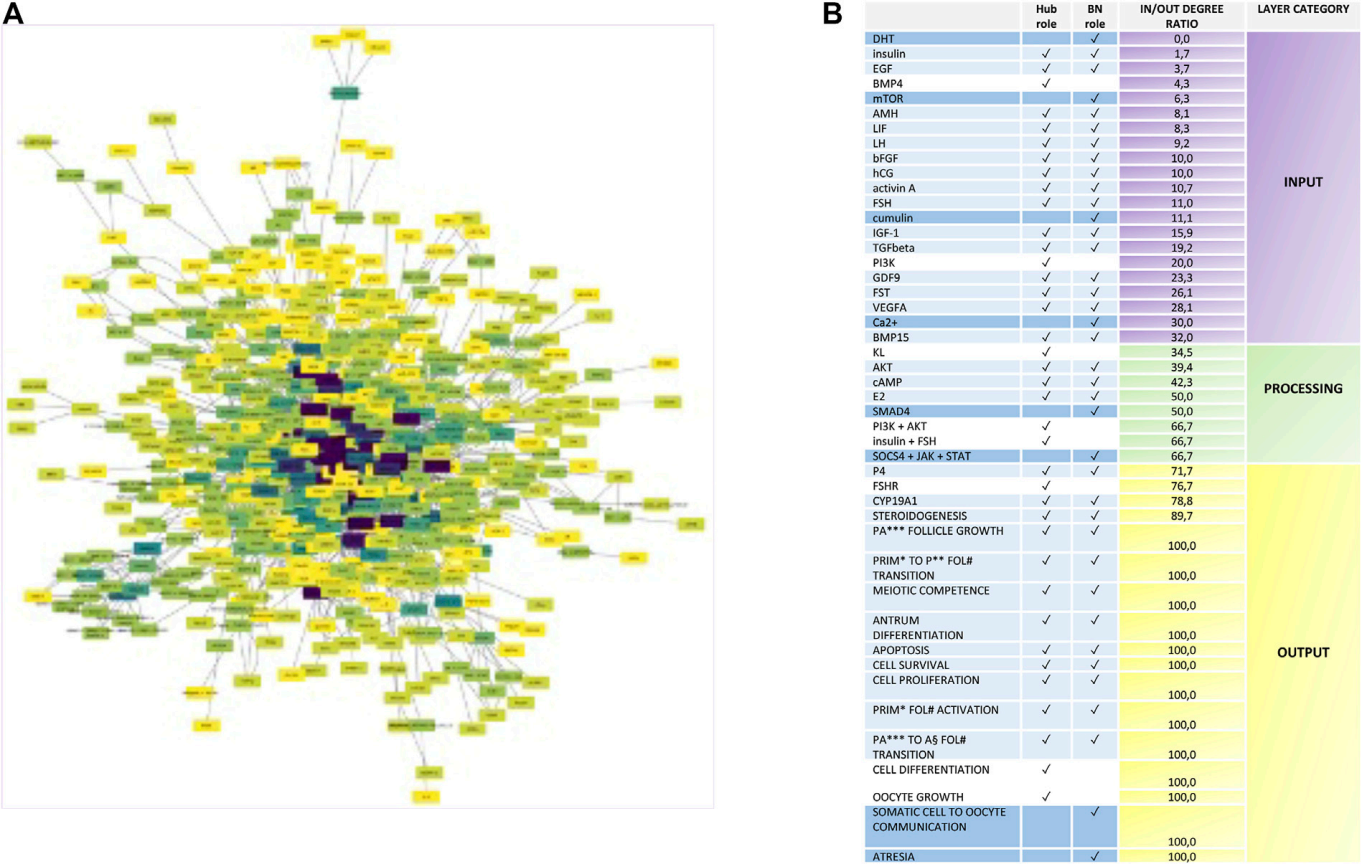
Nodes with Hub, bottleneck, and hub.BN role stratification into the ivF network. **(A)** Diagram showing the signal stratification of the ivF network. The color gradient varies depending on the direction of links characterizing each node, computed as in/out degree ratio, from purple **(higher)** to yellow **(lower)**. The spatial network arrangement was obtained by using the Cytoscape prefuse force-directed layout. **(B)** Node classification depending on their role in the signal propagation in the input layer (green scale color), processing layer (yellow scale color), and output layer (red scale color).

To organize the network depending on in/out connectivity, the in/out degree ratio of each node was computed (Figure 3A). This analysis allowed to stratify nodes in three layers (input, processing, and output layers) by classifying the network nodes on the basis of the in/out degree (Figure 3B):

DR_io_ 0–35 = input layer.

DR_io_ 36–67 = processing layer.

DR_io_ 68–100 = output layer.

The analysis displayed that the hub.BN was mostly abundant in the input layer (15 out of 30). BN mainly operated as processing (4 out of 8), whereas hubs were distributed in either input or output layers (3 out of 8, respectively).

The analysis of the *iv*F signaling network designed a coherent stratification of nodes by positioning in the input layer 16 out of 22 nodes belonging to the systemic endocrine controller released by pituitary (FSH and LH), chorion (hCG), and enteric-related endocrine glands (insulin, DHT, and IGF1) as well as reproductive controlling hormone/factors secreted either from follicles (FST, activin A, AMH, EGF, bFGF, TGFbeta, BMP4, and BMP15) or from oocytes (GDF9 and cumulin).

Analogously, the processing layer recognized among the eight nodes the intracellular second messenger cAMP and components of four signaling pathways: PI3K/AKT, JAK/STAT, TGFbeta (SMAD4), and KL (Figure 3B). Finally, 14 out of 17 nodes of the output layer were follicular events, and further three nodes were key components of steroidogenesis starting from the FSH receptor, the endpoint enzyme, and the hormone of follicular steroidogenesis (CYP19A1 and P4).

### Identification of New Molecular Players in the *iv*f Network Flow

**FIGURE 4:**
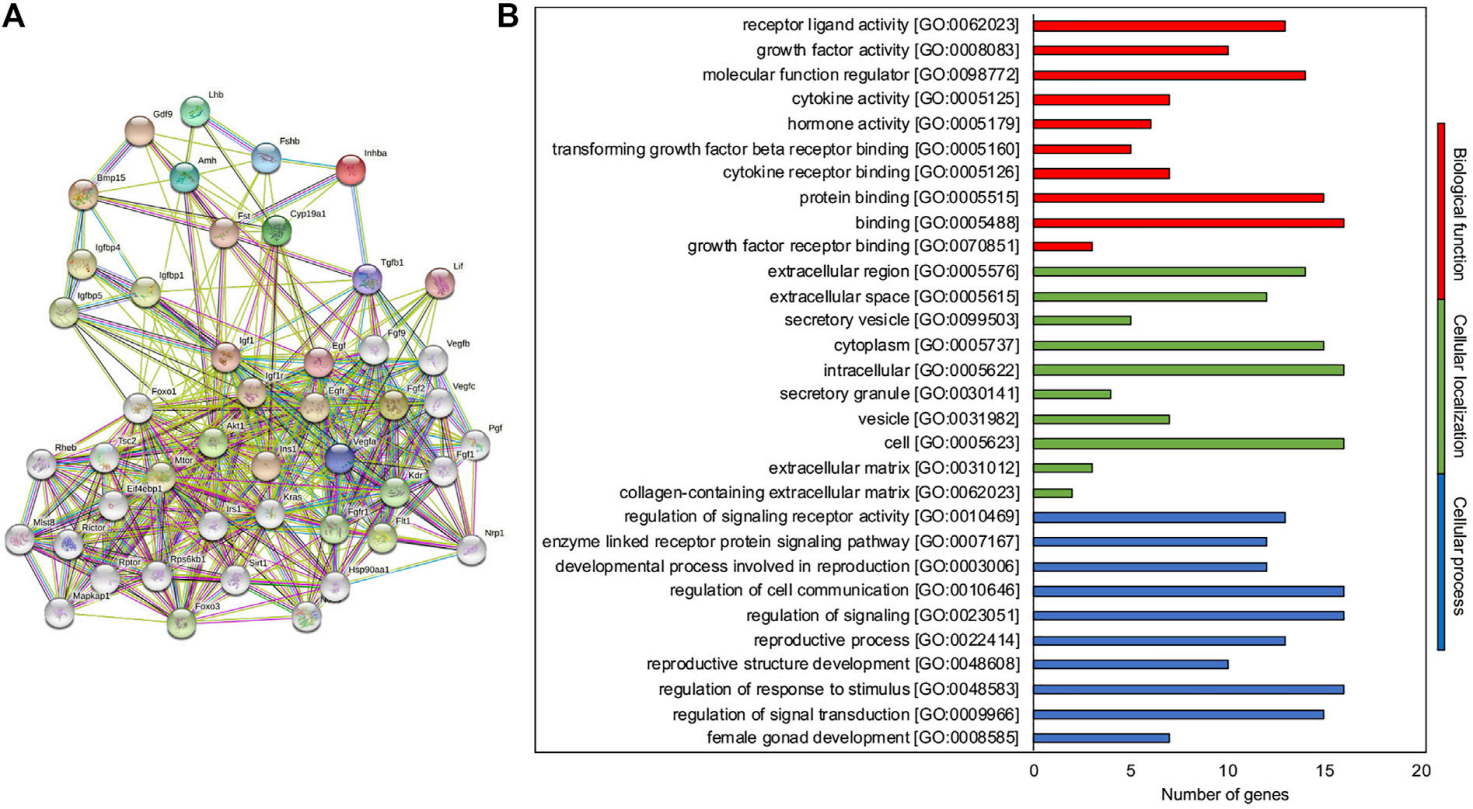
STRING-MMivF as the interaction network tuning the ivF process. **(A)** Known and predicted protein–protein interactions related to the hub.BN molecules of the network. The interactions include direct (physical) and indirect (functional) associations; they stem from computational prediction, from knowledge transfer between organisms, and from interactions aggregated from other (primary) databases. **(B)** Representative scheme of the top 10 most abundant GO terms identified for the new interactor molecules in the three GO categories: biological function (red), cellular localization (green), and biological processes (blue). The *x*-axis indicates the number of new interactor molecules in a specific category, while the *y*-axis indicates different GO terms.

To identify and predict new molecules involved in the *iv*F process, a functional protein association network (STRING_MM*iv*F) was created by STRING using as input hub.BN genes, where possible (Figure 4A). Totally, 30 new predicted interactor molecules were identified and summarized in Supplementary File 10.

Functional enrichment analysis was then performed (Supplementary File 11). The 10 most significantly enriched terms (*p* < 0.05) in each category are presented in Figure 4B. In the molecular function category, GO recognized the growth factor and intracellular signaling protein binding (7 out of 10) as the most abundant biological function regulating cellular processes such as metabolism control (2 out of 10), signal transduction (6 out of 10), and cell to cell communication (2 out of 10), sustaining angiogenesis. For the cellular component category, intracellular (3 out of 10), extracellular (2 out of 10), and mTOR complex (3 out of 10) localizations were reported accordingly.

Interestingly, involvement in follicular functions was found for most of the newly identified interactors, as reported in Supplementary File 10. Conversely, for four of them (KRAS, MLST8, MAPKAP1, and FGFR1), a specific role in the ovarian folliculogenesis has not been described to date. Of note, an abnormal female reproductive phenotype has been reported for KRAS and FGFR1 knock-in and knock-out mouse models, respectively (Table 3). Moreover, in the context of the STRING-MM*iv*F network, KRAS was predicted to interact with a high confidence score (score >0.9) with hub.BN AKT1, FGF2, and EGF but FGFR1 with hub.BN FGF2 (Table 3).

**TABLE 3:** Potential regulators of the ivF network.

Gene name	hub.BN interaction	STRING score	Defects in early folliculogenesis
MAPKAP1	AKT1	0.985	—
	IGF1	0.456	
KRAS	AKT1	0.949	Mice constitutively expressing KRASG12D in GC show impaired cell differentiation at the early stage of folliculogenesis, leading to the formation of abnormal follicle-like structures containing non-mitotic, non-apoptotic, and non-differentiated cells Fan et al. (2008)
	EGF	0.983	
	CYP19A1	0.465	
	VEGFA	0.820	
	FGF2	0.966	
	IGF1	0.703	
	TGFB1	0.497	
FGFR1	VEGFA	0.856	Hypomorphic mice show a short follicular phase with difficult entry into and termination of the luteal phase Tata et al. (2012)
	EGF	0.854	
	AKT1	0.448	
	FGF2	0.998	
	IGF1	0.801	
	INS	0.684	
MLST8	AKT1	0.973	—
	IGF1	0.471	

List of edges whose functions in the ovarian folliculogenesis have to be unveiled with annotations related to predicted links with hub.BN, STRING interaction score, and mouse phenotype related to defects in early phases of folliculogenesis.

## Discussion

The *iv*F network (Albert and Barabási, 2002) is characterized by a power-law distribution of node degree and by the absence of correlation between the node degree itself and the clustering coefficient in keeping with the Barabasi-Albert model, conferring to the network the following biologically relevant properties:

1) Robustness against random damage: when a random perturbation affects the network, it is very likely that only a scarcely linked node (i.e., a node belonging to the most frequent class of nodes) will be affected. Thus, the probability that a hub of the graph is affected remains very low, and in this model, it can be estimated to be about 5.9%.

2) Controllability: the small number of highly linked nodes implies that the whole system can be modulated with high efficiency by acting just on a few molecules, thus reducing the energetic cost and facilitating/accelerating the cell response.

3) Easy navigability: the virtual absence of clustering, together with the low values of the characteristic path length and of the average number of neighbors, confers to the network a typical structure of signaling networks. Interestingly, this low value of clustering coefficient also implies a little redundancy.

After having defined the topology, the computational analysis allowed to dissect the network to identify the *iv*F network flow (hub.BN). Indeed, the 2D KDE approach allowed to select those with a major modulatory role on the network information flow by identifying either cultural functional endpoints or *in vitro* follicle controllers such as systemic hormones and local factors (Matzuk et al., 2002).

More in detail, the priority of hub.BN was assigned by 2D KDE analysis to four events related to both follicle (primordial to primary follicle transition and preantral follicle growth) and oocyte development (oocyte meiotic competence). Furthermore, the analysis identified FSH as a key hub.BN; thus, reinforcing the idea of its central role as a controller of either the gonadotropin-dependent or independent phase of folliculogenesis (Holesh and Lord, 2018) (Casarini and Crépieux, 2019). Several pieces of scientific evidence collected mainly using *in vitro* studies, indeed, suggest the need to supplement FSH to also stimulate the early stage phases (from primary to later stages) (Hsueh et al., 2015) of folliculogenesis by stimulating either follicle growth or differentiation (Cecconi et al., 1999) (Barboni et al., 2011) (Cadoret et al., 2017) (Candelaria et al., 2020) (Cortvrindt et al., 1997) (Kreeger et al., 2005) (Wright et al., 1999). In addition, 7 hub.BN have been identified as main controllers of the network (activin A and LH, GDF-9, EGF, E2, Insulin, and IGF-1) (Orisaka et al., 2006) (Dong et al., 1996) (Edson et al., 2009).

The results of the network stratification analysis are in agreement with current knowledge on the physiological cross-talk between molecules regulating the inter- and intra-follicular communication. Indeed, in the input layer were identified hub.BN recapitulating the main modulatory factors, either hormones exerting a remote control on the ovary (LH, FSH, hCG, IGF1, and EGF) or molecules involved in intra-ovarian control of both somatic and germinal compartments (TGF-beta superfamily members and growth factors).

The processing layer is composed of hub.BN molecules belonging to PI3K/AKT, JAK/STAT, TGFbeta, and KL signaling pathways and the second messenger cAMP which transduce and amplify the actions of the previous endocrine and paracrine controllers (Li et al., 2008) (Fujihara et al., 2014) (John et al., 2009) (Imai et al., 2014).

Finally, the output layer recognized steroidogenic molecules (P4 and CYP19A1) which may be considered as endpoints of the pathways controlling *iv*F. The emerging picture defined by the computational stratification analysis thus supports the biological strength of the network recapitulating and organizing in three layers the physiological feedback orchestrating ovarian follicle development.

The biological strength of the network has been further confirmed by analyzing the reproductive phenotype of the available knock-out mice generated by using some *ivF* selected hub.BN. The analysis of the scientific evidence collected to date not only confirmed that the *in vivo* silencing of these controllers always promoted a negative impact on female fertility but also confirmed the interference specifically on pathways controlling the early phase of *in vivo* folliculogenesis, despite the different underlying mechanisms. STRING analysis was then performed to find new molecules and define their functional connections. Taking advantage of such an approach, molecules belonging to three effector categories have been identified as potential controllers of the early stage of the *in vitro* follicle development. Metabolic controllers involved in the signaling leading to follicle recruitment, apoptosis, and differentiation were identified. Indeed, STRING enriched the *iv*F network with mTOR and IGF1 signaling pathway molecules.

mTOR represents a molecular sensor for diverse environmental inputs including nutrients and growth factors and regulates various fundamental processes including cell growth, the metabolism, differentiation, and autophagy (Long et al., 2003). In folliculogenesis, some lines of evidence have shown that the IGF1-dependent activation (hub.BN role in the *iv*F network) of the PI3K/AKT signaling (hub role in the *iv*F network) and its downstream cognate mTOR complex sustains ovarian primordial follicle dormancy and activation, oocyte maintenance and activation, and GC proliferation and differentiation (Makker et al., 2014) (Guo and Yu, 2019) (Herta et al., 2018).

More in detail, mTORC1 plays a documented role in primordial follicle-oocyte bidirectional signaling. It was shown to activate the KIT receptor in oocytes through the KIT ligand (hub role in the *iv*F network), which triggers a PI3K/PTEN/AKT/forkhead box 3 (FOXO3) cascade and awakens the dormant oocytes (Zhao et al., 2018) (Liu et al., 2014). In the awakened oocyte, secretion of oocyte-specific growth factors such as BMP15 (hub.BN role in the *iv*F network) and GDF9 (hub.BN in the *iv*F network) further activates receptor serine kinases and downstream SMAD (mothers against DPP homolog 1 Drosophila) proteins including SMAD4 (BN role in the *iv*F network) in surrounding GC, leading to their growth and proliferation (Sanfins et al., 2018). A targeted deletion of the mTORC1 negative regulator TSC2 (STRING-enriched partner) in mouse oocytes results in prematurely follicular activation due to elevated mTORC1 activity in oocytes which in turn cause depletion of follicles in the early adulthood (Adhikari and Liu, 2009). Of note, a compensatory elevation of PI3K signaling was proposed to the reason for the unaffected follicular development observed in RPTOR (STRING-enriched partner) conditional KO in primordial and all subsequent oocyte stages (Gorre et al., 2014). Conversely, conditional KO mice for RPTOR in primordial follicle GC prevent the cell differentiation, and this arrests the dormant oocytes in their quiescent states, leading to the oocyte death age (Liu et al., 2014), indicating that the KIT/PI3K cascade in oocytes is indispensable for primordial follicle survival.

The key role of mTOR signaling was also supported by the computational identification of the downstream target of mTOR, such as FOXO1, FOXO3, and SIRT1 (STRING-enriched partners). Furthermore, transgenic mice for these molecules have provided evidence on their cross-talk in regulating the dynamics of the primordial follicle pool (Adhikari and Liu, 2009) (Adhikari et al., 2013) (Chen et al., 2015).

FOXO3 functions at the earliest stages of follicular growth as a suppressor of follicular overactivation, increasing the follicle reserves in the ovary in order to extend the reproductive period of females (Castrillon et al., 2003) (Shah et al., 2018) (Lee and Chang, 2019). Accordingly, FOXO3 has been found to be highly expressed in the nuclei of oocytes of primordial follicles, and its expression is downregulated in oocytes of primary and later-growing follicles, indicating that its downregulation in oocytes could be a prerequisite for the initiation of oocyte growth during follicular activation (Liu et al., 2007).

Furthermore, FOXO3−/− female mice exhibit a distinctive ovarian phenotype of global follicular activation, leading to oocyte death, early depletion of functional ovarian follicles, and secondary infertility (Castrillon et al., 2003) (Hosaka et al., 2004) (Lin et al., 2004).

Of note, selective depletion of FOXO1 and FOXO3 in mouse GC leads to an infertile phenotype characterized by metabolic changes and the production of factors that exerts potent negative feedback to prevent gene expression of pituitary FSH (hub.BN role in the *iv*F network). Decreased levels of serum FSH further restrict follicle growth and development, ultimately preventing ovulation. Besides, FOXO1/3 depletion alters the expression of genes involved in follicle growth and apoptosis, disrupting cell regulatory signals associated with the granulosa cell metabolism and follicle growth. These results reveal a novel ovarian-pituitary endocrine feedback loop preventing uncontrolled proliferation and/or premature differentiation of GC in follicles where apoptosis is impaired (Liu Z. et al., 2013).

Accordingly, the STRING-enriched molecule FOXO1 was shown to act as a silent guardian of follicle development. Indeed, it is a critical factor in promoting GC apoptosis to counteract the stimulatory FSH role, one of the selected *ivF* network hub.BN (Liu et al., 2009) (Fan et al., 2010) (Shen et al., 2014). More in detail, FSH was found to promote the expression of GC genes required for proliferation, survival, and estrogen synthesis by decreasing FOXO1, which negatively regulates proliferation and steroidogenesis (Liu et al., 2009) (Fan et al., 2010) (Shen et al., 2014) (Rosairo et al., 2008).

Moreover, the identification of SIRT1 as a potential partner of STRING enhances the role of the mTOR pathway in the control of *in vitro* early mammalian folliculogenesis. Indeed, it has been proposed as the molecular mediator of the calorie restriction-dependent prevention of follicular activation, leading to ovarian reserve preservation (Bordone et al., 2007) (Long et al., 2019). At a molecular level, the effect of SIRT1 on the ovary occurs through the deacetylation dependent-activation of FOXO3 and the suppression of the mTOR signaling (Long et al., 2019). The latter could be mediated by TSC2-SIRT1 interactions (Ghosh et al., 2010). These results are in good agreement with data showing that the SIRT1 activator (SRT1720) improves the follicle reserve and prolongs the ovarian lifespan of diet-induced obesity in female mice via activating SIRT1 and suppressing mTOR signaling (Zhou et al., 2014). Conversely, a recent work shows how SIRT1 can sustain the activation of mouse primordial follicles independent of its deacetylase activity. Specifically, SIRT1 was shown to trigger primordial follicle awakening by activating the PI3K/AKT-AKT and TSC1/2-mTOR signaling pathways. Indeed, its pharmacologically (resveratrol) and/or genetic-induced activation in mouse ovary cultures leads to SIRT1 to work as a transcription cofactor which modulates the expression levels of AKT, mTOR, and genes related to classic primordial follicle activation (Zhang et al., 2019). Interestingly, SIRT1 overactivation was shown to inhibit FOXO3 activity by promoting its exclusion form the nucleus through the AKT-dependent phosphorylation (Zhang et al., 2019), suggesting that SIRT1 activators might be used to efficiently activate the primordial follicles to be applied in *in vitro* activation protocols and/or to avoid uncontrolled follicular atresia characterizing POF.

The evidence of mTOR and SIRT1 pathways on follicular fate has been achieved by taking advantage of their *in vivo* and *in vitro* pharmacological modulation.

*In vivo*, the SIRT1 activation has been achieved by caloric restriction or the application of specific sirtuin activators or mTOR blockers. Caloric restriction was shown to induce the accumulation of SIRT1 in murine ovaries, which is associated with inhibition of primordial follicle activation and impairment of ovarian follicle development (Tatone et al., 2015) (Liu et al., 2015) (Long et al., 2019) mechanisms probably involved in mice delay of puberty, prolongation of reproductive lifespan, and prevention of age-associated infertility (Liu M. et al., 2013).

Various effects on folliculogenesis have been observed *in vitro* by using the SIRT1 activator, mTOR inhibitor, and resveratrol.

More in detail, it promoted growth in human ovarian follicles (Hao et al., 2018) and in ovine primordial follicles (Bezerra et al., 2018). The resveratrol action is addressed to regulate GC cell proliferation and survival (Han et al., 2017) (Ortega et al., 2012) (Schube et al., 2014) (Wong et al., 2010) as well as steroidogenesis (Qasem, 2020) (Morita et al., 2012) even if with contradictory results.

In late folliculogenesis, mTOR signaling is dependent on gonadotropin FSH modulation to manage processes such as oocyte maturation (Guo et al., 2018), ovarian somatic cell proliferation, and steroidogenesis (Palaniappan and Menon, 2012) and to intensify EGFR/RAS signaling (Fan et al., 2009) (Gloaguen et al., 2011) (Fan et al., 2012) (Wayne et al., 2007).

Interestingly, the STRING network enrichment identified the isoform KRAS as a potential interactor of the AKT1 and EGF hub.BN molecules with a high confidence score, suggesting its key role as a metabolic effector of gonadotropin stimulation. Its contribution in follicular development is corroborated using conditional knock-in mouse models in which the GC expresses a constitutively active form of KRAS (KrasG12D). These models show alterations in GC differentiation, proliferation, and apoptosis at early stage of folliculogenesis, thus impairing cell responses to gonadotropins and leading to premature ovarian failure (Fan et al., 2008) (Fan and Richards, 2010) (Bulun et al., 2019). This phenotype is similar to that of the mutant mouse model with EGFR signaling defects (Hsieh et al., 2007). The altered response of KRASG12D-expressing GC to gonadotropins appears to be related to low levels of FSHR and the inability of FSH to induce expression of LHCGR mRNA and therefore to the loss of the crucial LH-MAPK3/1 signaling pathways. This conclusion is supported by the reduced expression of specific genes known to be essential for COC expansion and ovulation (Richards, 2005). Moreover, mutations causing KRAS overactivity have been reported as markers of epithelial and endometrioid ovarian cancer (Ramalingam, 2016) (Dinulescu et al., 2005).

Although the effect of these energy sensors in folliculogenesis remains to be investigated in depth, some data have been reported in the literature.

The third category of molecules enriched from the STRING network was represented by several angiogenetic factors. Indeed, it must be considered that as the follicle growth, the mTOR-dependent metabolic signaling needs to be implemented with components of the vascular system that guarantees the correct trophic supply and spreading of precursors to properly complete folliculogenesis (Martelli et al., 2017).

The angiogenesis process assumes a key role in the contest of the reproductive function (Robinson et al., 2009) (Wulff et al., 2002). Accordingly, angiogenesis inhibition leads to the reduction of follicular growth, ovulation disruption, and drastic effects on the corpus luteum activity and development (Robinson et al., 2009). The large amount of factors is able to control angiogenesis and their spatio-temporal regulatory activities, suggesting that more than one factor might be useful for angiogenesis well-functioning associated with ovulation.

Specifically, functional interactions from STRING were unveiled for VEGF members (VEGFA, VEGFB, VEGFC, and VEGFD), VEGF receptors (NRP1, KDR, and FLT), and angiogenic factors (FGF1, FGF9, PGF, and FGFR).

Several pieces of evidence confirmed that both somatic and germinal compartments contribute to modulate the expression of VEGFA by modulating the blood vessel network during preantral follicle development. More in detail, small and middle pig preantral follicles seem to behave as autonomous recruited units, where the growth of the somatic compartment is always accompanied by the simultaneous activation of the endothelial cells (Martelli et al., 2017).

Differently, once the preantral follicles reach the late stage enclosing an almost fully growth oocyte, it starts to express high and stable levels of VEGFA, essential to maintain follicle angiogenesis in a steady status of activation (Martelli et al., 2017). Furthermore, high levels of VEGFA also characterize the preantral follicular structures at the stage of antrum formation. In this context, VEGFA has been supposed to increase microvessel permeability by stimulating plasma extravasations, thus allowing the accumulation of fluids within the differentiating follicular cavity (Isobe et al., 2005).

The strict correlation between somatic and vascular parameters may represent an indirect biological validation of the key role of VEGF members identified by *iv*F network STRING analysis depicting a synergic action between follicle compartments and blood vessel system components as a key event driving preantral follicle activation first and then sustaining the process of transition from preantral to early antral follicles (Martelli et al., 2017) (Danforth et al., 2003) (Abramovich et al., 2006).

These molecules exert their function through their receptors (Stouffer et al., 2001) and are tightly regulated by paracrine factors such as nitric oxide. Nitric oxide has been reported to mediate positive effects on follicle development and selection related to angiogenic events and play a modulatory role in the ovarian steroidogenesis (Basini and Grasselli, 2015).

Nevertheless, proangiogenetic factors such as FGF1, FGF9, and PGF have also been described as they can potentiate the effect of VEGF signaling by increasing the VEGF expression in theca cells of cattle (Gray et al., 1987) (Gospodarowicz et al., 1987) (Berisha et al., 2004).

In conclusion, the computational analysis of the *iv*F network has enabled us to select among several molecules adopted to date those that are the main spatio-temporal controllers of the early stage of follicle development. Taking advantage of the biological robustness of such a network, new molecules slightly deepened or unexplored for *iv*F purposes were identified by taking advantage of the STRING approach. Altogether, this evidence suggests the *iv*F network as a sounded system biology tool to be exploited for research, technological, and innovation aims with the final goal of designing new diagnostic and therapeutic strategies for female fertility.

## Data Availability

The original contributions presented in the study are included in the article/Supplementary Material, and further inquiries can be directed to the corresponding author.
