# Supplementary material for: In Vitro Folliculogenesis in Mammalian Models: A Computational Biology Study
Source: Front Mol Biosci. 2021 Nov 9;8:737912. doi: 10.3389/fmolb.2021.737912 (PMC8630647; doi:10.3389/fmolb.2021.737912)
Supplement: Supplementary file 1 [file DataSheet1.ZIP › SUPPL FILES Frontiers Mol Bio/Suppl File 7.docx]

Supplementary Material

Supplementary File 7

|  | **Hub role** | **BN role** | **Ovarian functional annotation** | **Gene cards annotation for gene-derived molecules** | | |
| --- | --- | --- | --- | --- | --- | --- |
| APOPTOSIS | **✓** | **✓** |  |  |  |  |
| MEIOTIC COMPETENCE | **✓** | **✓** |  |  |  |  |
| CELL PROLIFERATION | **✓** | **✓** |  |  |  |  |
| VEGF-A | **✓** | **✓** | Endothelial cell-derived growth factor working as a key regulator of angiogenesis, promoting ovarian follicular development and transition from the primary to the secondary follicle stage [1] | [VEGFA](https://www.genecards.org/cgi-bin/carddisp.pl?gene=VEGFA&keywords=VEGFA) |  |  |
| bFGF | **✓** | **✓** | Granulosa and/or theca cell-derived growth factor (depending on the specie) regulating early follicle development [2] | [FGF2](https://www.genecards.org/cgi-bin/carddisp.pl?gene=FGF2&keywords=FGF2) |  |  |
| PA*** FOLLICLE GROWTH | **✓** | **✓** |  |  |  |  |
| EGF | **✓** | **✓** | Growth hormone derived from granulosa cells compartment, involved in the regulation of oocyte development [3] | [EGF](https://www.genecards.org/cgi-bin/carddisp.pl?gene=EGF&keywords=EGF) |  |  |
| cAMP | **✓** | **✓** | Ubiquitous second messenger molecule regulated in many physiological processes. In the ovary it plays an essential role in maintaining oocyte meiotic arrest [4][5] |  |  |  |
| PRIM* FOL# ACTIVATION | **✓** | **✓** |  |  |  |  |
| AKT | **✓** | **✓** | Ubiquitous serine/threonine-protein kinase implicated in the regulation of primordial follicle activation and growth. Also involved in oocyte meiotic resumption and spindle organization [6] | [AKT1](https://www.genecards.org/cgi-bin/carddisp.pl?gene=AKT1&keywords=AKT1) |  |  |
| FSH | **✓** | **✓** | Hypophysis-derived peptide hormone controlling ovarian follicle functions [7] | [FSHB](https://www.genecards.org/cgi-bin/carddisp.pl?gene=FSHB&keywords=fshb) |  |  |
| E2 | **✓** | **✓** | Estrogen steroid hormone produced especially within the ovarian follicles, widely implicated in the regulation of the menstrual and estrus cycle [8] |  |  |  |
| FST | **✓** | **✓** | Hypophysis-derived protein mainly expressed in granulosa cells. It inhibits the biosynthesis and secretion of pituitary FSH [9] | [FST](https://www.genecards.org/cgi-bin/carddisp.pl?gene=FST&keywords=FST) |  |  |
| P4 | **✓** | **✓** | Steroid and progestogen sex hormone produced by ovaries, placenta and adrenal glands. It is involved in the menstrual cycle, pregnancy and embryogenesis [10] |  |  |  |
| ANTRUM DIFFERENTIATION | **✓** | **✓** |  |  |  |  |
| GDF9 | **✓** | **✓** | Oocyte-secreted growth differentiation factor required for the ovarian folliculogenesis. Promotes primordial follicle development and stimulates granulosa cell proliferation [11] | [GDF9](https://www.genecards.org/cgi-bin/carddisp.pl?gene=GDF9&keywords=gdf9) |  |  |
| CYP19A1 | **✓** | **✓** | Monooxygenase catalyzing the last step of estrogen biosynthesis. Its activity (expression) varies among different cell types depending on the cells' need for estrogen. Mostly active in the ovary where it guides sexual development [12] | [CYP19A1](https://www.genecards.org/cgi-bin/carddisp.pl?gene=CYP19A1&keywords=CYP19A1) |  |  |
| BMP15 | **✓** | **✓** | Oocyte-secreted ligand of the TGFbeta family playing a role in oocyte maturation and follicular development through activation of granulosa cells [11] | [BMP15](https://www.genecards.org/cgi-bin/carddisp.pl?gene=BMP15&keywords=BMP15) |  |  |
| activin A | **✓** | **✓** | Ovarian glycoprotein dimer mainly produced by granulosa cells which exerts either local or central role by activating FSH secretion [13] | [INHBA](https://www.genecards.org/cgi-bin/carddisp.pl?gene=INHBA&keywords=INHBA) |  |  |
| IGF1 | **✓** | **✓** | Peptide growth hormone produced primary by the liver. It participates in the regulation of puberty and fertility as a primary mediator of the growth hormone GH [14] | [IGF1](https://www.genecards.org/cgi-bin/carddisp.pl?gene=IGF1&keywords=IGF1) |  |  |
| LH | **✓** | **✓** | Hypophysis-derived glycoprotein hormone controlling ovulation by stimulating the ovaries to synthesize steroids [15] | [LHB](https://www.genecards.org/cgi-bin/carddisp.pl?gene=LHB&keywords=LHB) |  |  |
| AMH | **✓** | **✓** | Dimeric glycoprotein produced by granulosa cells of the preantral and small antral ovarian follicles which plays a role in follicular development in adult females [16] | [AMH](https://www.genecards.org/cgi-bin/carddisp.pl?gene=AMH&keywords=AMH) |  |  |
| hCG | **✓** | **✓** | Peptide hormone synthesized by pre-embryonic cells (syncytiotrophoblast) stimulating the ovaries to synthesize the steroids that are essential for the maintenance of pregnancy [17] | [CGB1](https://www.genecards.org/cgi-bin/carddisp.pl?gene=CGB1&keywords=CGB) |  |  |
| LIF | **✓** | **✓** | Pleiotropic cytokine mainly expressed in granulosa cells. It is involved in the early follicular phases such as primordial follicle activation [18] | [LIF](https://www.genecards.org/cgi-bin/carddisp.pl?gene=LIF&keywords=LIF) |  |  |
| PRIM* TO P** FOL# TRANSITION | **✓** | **✓** |  |  |  |  |
| CELL SURVIVAL | **✓** | **✓** |  |  |  |  |
| STEROIDOGENESIS | **✓** | **✓** |  |  |  |  |
| TGFbeta | **✓** | **✓** | Ubiquitous growth factor. In the ovary it is expressed by granulosa cells, theca cells and oocyte in a developmental, stage-related manner, working as intraovarian regulator of folliculogenesis [19] | [TGFB1](https://www.genecards.org/cgi-bin/carddisp.pl?gene=TGFB1&keywords=TGFB1) |  |  |
| insulin | **✓** | **✓** | Peptide hormone with anabolic properties, produced by the pancreatic beta cells. It stimulates proliferation and steroidogenesis in granulosa and theca cells [20] | [INS](https://www.genecards.org/cgi-bin/carddisp.pl?gene=INS&keywords=INS) |  |  |
| PA*** TO A§ FOL# TRANSITION | **✓** | **✓** |  |  |  |  |
| insulin + FSH | **✓** |  | Their synergic action is more efficient in promoting resumption of oocyte meiosis, maintaining survival and stimulating follicular development [21][20] | [INS](https://www.genecards.org/cgi-bin/carddisp.pl?gene=INS&keywords=INS) | [FSHB](https://www.genecards.org/cgi-bin/carddisp.pl?gene=FSHB&keywords=fshb) |  |
| CELL DIFFERENTIATION | **✓** |  |  |  |  |  |
| FSHR | **✓** |  | Receptor for FSH playing a key role in gonad development. It is expressed in granulosa cells [7] | [FSHR](https://www.genecards.org/cgi-bin/carddisp.pl?gene=FSHR&keywords=FSHR) |  |  |
| OOCYTE GROWTH | **✓** |  |  |  |  |  |
| PI3K + AKT | **✓** |  | Signaling cascade with a critical role in the regulation of primordial follicle survival and activation [6] | [PI3KCA](https://www.genecards.org/cgi-bin/carddisp.pl?gene=PIK3CA&keywords=PI3KCA) | [AKT1](https://www.genecards.org/cgi-bin/carddisp.pl?gene=AKT1&keywords=AKT1) |  |
| KL | **✓** |  | Granulosa factor with pleiotropic actions in the control of oocyte and follicle growth [22] | [KITLG](https://www.genecards.org/cgi-bin/carddisp.pl?gene=KITLG&keywords=KITLG) |  |  |
| PI3K | **✓** |  | Ubiquitous protein kinase generating PIP3, a key mediator of signaling cascades promoting oocyte growth and early follicular development [23] | [PI3KCA](https://www.genecards.org/cgi-bin/carddisp.pl?gene=PIK3CA&keywords=PI3KCA) |  |  |
| BMP4 | **✓** |  | Stromal and theca cell-secreted ligand of the TGFbeta family acting as a positive regulator of the primordial to primary follicle transition [24] | [BMP4](https://www.genecards.org/cgi-bin/carddisp.pl?gene=BMP4&keywords=BMP4) |  |  |
| SOCS4 + JAK + STAT |  | **✓** | The JAK1/STAT3 pathway activation is positively involved in primordial follicle activation. The transcription and translation of SOCS4, responsible for the regulation of JAK/STAT signaling, is implicated in the follicular development control and in the regulation of mammalian female lifespan, besides the delaying of reproductive senescence onset [25] | [SOCS4](https://www.genecards.org/cgi-bin/carddisp.pl?gene=SOCS4&keywords=SOCS4) | [JAK1](https://www.genecards.org/cgi-bin/carddisp.pl?gene=JAK1&keywords=JAK) | [STAT1](https://www.genecards.org/cgi-bin/carddisp.pl?gene=STAT1&keywords=stat) |
| cumulin |  | **✓** | Oocyte-derived heterodimer of the TGFbeta family. It is a potent activator of granulosa cells and improves oocyte quality [26][27] |  |  |  |
| SOMATIC CELL TO OOCYTE COMMUNICATION |  | **✓** |  |  |  |  |
| mTOR |  | **✓** | Serine/threonine protein kinase involved in several aspects of follicular development, oocyte genome integrity and competence. It is expressed in granulosa cells and in the oocyte [28][29] | MTOR |  |  |
| Ca2+ |  | **✓** | Oocyte-produced secondary messenger within the ovary. It regulates different metabolic pathways and cellular processes during oocyte activation [30] |  |  |  |
| ATRESIA |  | **✓** |  |  |  |  |
| DHT |  | **✓** | Androgen sex steroid produced primary by the liver. It affects folliculogenesis directly via androgen receptors or indirectly through aromatization to estrogen [31][32] |  |  |  |
| SMAD4 |  | **✓** | SMAD4 is expressed in both prepubertal and pubertal ovaries, with localization in all follicular stages, suggesting a key role as regulator of follicle development and oocyte growth [33] |  |  |  |

**Suppl File 7. Node classification.** Nodes were grouped as hubs, BN and hub.BN. For each node the main biological function in the ovarian context has been reported. The table also shows topological features identified from the computational analysis and the link to the Gene cards database (<https://www.genecards.org>) providing comprehensive information on all annotated and predicted genes. The knowledgebase automatically integrates gene-centric data from ~150 web sources, including genomic, transcriptomic, proteomic, genetic, clinical and functional information (PRIM* primordial, P** Primary, PA*** preantral, A§ antral, and Fol# Follicle).

**References:**

1. Araújo VR, Gastal MO, Figueiredo JR, Gastal EL (2014) In vitro culture of bovine preantral follicles: a review. Reprod Biol Endocrinol 12:78. https://doi.org/10.1186/1477-7827-12-78

2. Nilsson E, Parrott JA, Skinner MK (2001) Basic fibroblast growth factor induces primordial follicle development and initiates folliculogenesis. Mol Cell Endocrinol. https://doi.org/10.1016/S0303-7207(01)00391-4

3. Richani D, Gilchrist RB (2018) The epidermal growth factor network: Role in oocyte growth, maturation and developmental competence. Hum Reprod Update. https://doi.org/10.1093/humupd/dmx029

4. Pan B, Li J (2019) The art of oocyte meiotic arrest regulation. Reprod Biol Endocrinol. https://doi.org/10.1186/s12958-018-0445-8

5. Heiligentag M, Eichenlaub-Ritter U (2018) Preantral follicle culture and oocyte quality. Reprod Fertil Dev 30:18–43. https://doi.org/10.1071/RD17411

6. Sobinoff AP, Sutherland JM, Mclaughlin EA (2013) Intracellular signalling during female gametogenesis. Mol. Hum. Reprod.

7. Casarini L, Crépieux P (2019) Molecular mechanisms of action of FSH. Front. Endocrinol. (Lausanne).

8. Reed BG, Carr BR (2000) The Normal Menstrual Cycle and the Control of Ovulation

9. Das N, Kumar TR (2018) Molecular regulation of follicle-stimulating hormone synthesis, secretion and action. J. Mol. Endocrinol.

10. Cable JK, Grider MH (2020) Physiology, Progesterone

11. Belli M, Shimasaki S (2018) Molecular Aspects and Clinical Relevance of GDF9 and BMP15 in Ovarian Function. In: Vitamins and Hormones

12. Stocco C (2008) Aromatase expression in the ovary: Hormonal and molecular regulation. Steroids

13. Tanaka Y (2019) 165-171, February, 2019

14. Devesa J, Caicedo D (2019) The role of growth hormone on ovarian functioning and ovarian angiogenesis. Front. Endocrinol. (Lausanne).

15. Nedresky D, Singh G (2019) Physiology, Luteinizing Hormone

16. Ozzola G (2017) Anti-Müllerian hormone: A brief review of the literature. Clin Ter. https://doi.org/10.7417/CT.2017.1976

17. Nwabuobi C, Arlier S, Schatz F, et al (2017) hCG: Biological functions and clinical applications. Int. J. Mol. Sci.

18. Nilsson EE, Kezele P, Skinner MK (2002) Leukemia inhibitory factor (LIF) promotes the primordial to primary follicle transition in rat ovaries. Mol Cell Endocrinol. https://doi.org/10.1016/S0303-7207(01)00746-8

19. McGee EA, Raj RS (2015) Regulators of ovarian preantral follicle development. Semin Reprod Med 33:179–184. https://doi.org/10.1055/s-0035-1552584

20. Rossetto R, Saraiva MVA, Bernuci MP, et al (2016) Impact of insulin concentration and mode of FSH addition on the in vitro survival and development of isolated bovine preantral follicles. Theriogenology 86:1137–1145. https://doi.org/10.1016/j.theriogenology.2016.04.003

21. Chaves RN, Duarte ABG, Rodrigues GQ, et al (2012) The Effects of Insulin and Follicle-Simulating Hormone (FSH) During In Vitro Development of Ovarian Goat Preantral Follicles and the Relative mRNA Expression for Insulin and FSH Receptors and Cytochrome P450 Aromatase in Cultured Follicles1. Biol Reprod 87:1–11. https://doi.org/10.1095/biolreprod.112.099010

22. Driancourt MA, Reynaud K, Cortvrindt R, Smitz J (2000) Roles of KIT and KIT LIGAND in ovarian function. Rev Reprod 5:143–152. https://doi.org/10.1530/ror.0.0050143

23. McLaughlin M, Innell HL, Anderson RA, Telfer EE (2014) Inhibition of phosphatase and tensin homologue (PTEN) in human ovary in vitro results in increased activation of primordial follicles but compromises development of growing follicles. Mol Hum Reprod 20:736–744. https://doi.org/10.1093/molehr/gau037

24. Knight PG, Glister C (2006) TGF-β superfamily members and ovarian follicle development. Reproduction

25. Sutherland JM, Keightley RA, Nixon B, et al (2012) Suppressor of cytokine signaling 4 (SOCS4): Moderator of ovarian primordial follicle activation. J Cell Physiol. https://doi.org/10.1002/jcp.22837

26. Mottershead DG, Sugimura S, Al-Musawi SL, et al (2015) Cumulin, an oocyte-secreted heterodimer of the transforming growth factor-β family, is a potent activator of granulosa cells and improves oocyte quality. J Biol Chem. https://doi.org/10.1074/jbc.M115.671487

27. Richani D, Constance K, Lien S, et al (2019) Cumulin and FSH cooperate to regulate inhibin B and activin B production by human granulosa-lutein cells in vitro. Endocrinology. https://doi.org/10.1210/en.2018-01026

28. Guo Z, Yu Q (2019) Role of mTOR Signaling in Female Reproduction. Front. Endocrinol. (Lausanne).

29. Guo J, Zhang T, Guo Y, et al (2018) Oocyte stage-specific effects of MTOR determine granulosa cell fate and oocyte quality in mice. Proc Natl Acad Sci U S A. https://doi.org/10.1073/pnas.1800352115

30. Stewart TA, Davis FM (2019) An element for development: Calcium signaling in mammalian reproduction and development. Biochim. Biophys. Acta - Mol. Cell Res.

31. Gervásio CG, Bernuci MP, Silva-de-Sá MF, Rosa-e-Silva ACJ de S (2014) The Role of Androgen Hormones in Early Follicular Development. ISRN Obstet Gynecol. https://doi.org/10.1155/2014/818010

32. Walters KA (2015) Role of androgens in normal and pathological ovarian function. Reproduction

33. Xing N, Liang Y, Gao Z, et al (2014) Expression and localization of Smad2 and Smad4 proteins in the porcine ovary. Acta Histochem 116:1301–1306. https://doi.org/10.1016/j.acthis.2014.07.014
