# Supplementary material for: In Vitro Folliculogenesis in Mammalian Models: A Computational Biology Study
Source: Front Mol Biosci. 2021 Nov 9;8:737912. doi: 10.3389/fmolb.2021.737912 (PMC8630647; doi:10.3389/fmolb.2021.737912)
Supplement: Supplementary file 1 [file DataSheet1.ZIP › SUPPL FILES Frontiers Mol Bio/Suppl File 6.pptx]

## Slide 1
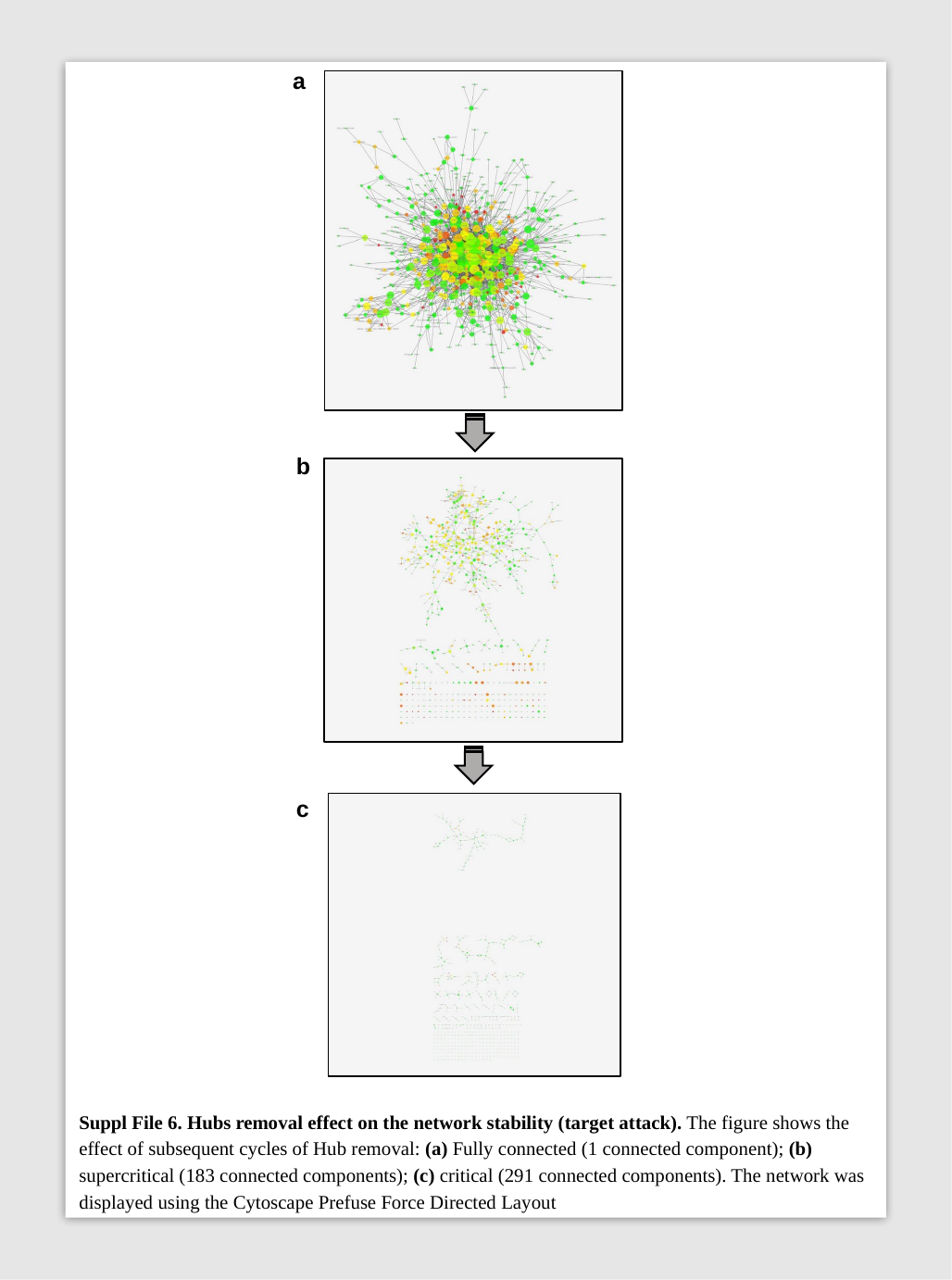

a
b
2
3
1
c
Suppl File 6. Hubs removal effect on the network stability (target attack). The figure shows the effect of subsequent cycles of Hub removal: (a) Fully connected (1 connected component); (b) supercritical (183 connected components); (c) critical (291 connected components). The network was displayed using the Cytoscape Prefuse Force Directed Layout
