# Supplementary material for: In Vitro Folliculogenesis in Mammalian Models: A Computational Biology Study
Source: Front Mol Biosci. 2021 Nov 9;8:737912. doi: 10.3389/fmolb.2021.737912 (PMC8630647; doi:10.3389/fmolb.2021.737912)
Supplement: Supplementary file 1 [file DataSheet1.ZIP › SUPPL FILES Frontiers Mol Bio/Suppl File 10.docx]

Supplementary Material

Supplementary File 10

| **N°** | **Interactor gene name** | **Ovarian functional annotation** | **Gene cards annotation** |
| --- | --- | --- | --- |
| **1** | **KRAS** | Proto-oncogene with GTPase activity. Frequently mutated in major types of epithelial ovarian carcinomas [1][2] | [KRAS](https://www.genecards.org/cgi-bin/carddisp.pl?gene=KRAS&keywords=kras) |
| **2** | **EGFR** | Tyrosine kinase receptor binding EGF. It is involved in oocyte maturation, cumulus expansion and ovulation. It is mainly expressed in granulosa cells [3] | [EGFR](https://www.genecards.org/cgi-bin/carddisp.pl?gene=EGFR&keywords=egf) |
| **3** | **IRS1** | Insulin receptor tyrosine kinase substrate that acts as a downstream target of the FSH and IGF1 cascades. It activates PI3K/AKT pathway promoting follicle maturation. Mainly expressed in granulosa cells [4] | [IRS1](https://www.genecards.org/cgi-bin/carddisp.pl?gene=IRS1&keywords=IRS1) |
| **4** | **IGFBP1** | Insulin-like growth factor binding protein. It has been shown to either inhibit or stimulate the growth promoting effects of the IGF during follicular development. Mainly expressed in granulosa cells [5][6] | [IGFBP1](https://www.genecards.org/cgi-bin/carddisp.pl?gene=IGFBP1&keywords=IGFBP1) |
| **5** | **IGFBP4** | Insulin-like growth factor binding protein. It has been shown to either inhibit or stimulate the growth promoting effects of the IGF during follicular development. Mainly expressed in granulosa cells [5][6] | [IGFBP4](https://www.genecards.org/cgi-bin/carddisp.pl?gene=IGFBP4&keywords=IGFBP4) |
| **6** | **IGFBP5** | Insulin-like growth factor binding protein. It has been shown to either inhibit or stimulate the growth promoting effects of the IGF during follicular development. Mainly expressed in granulosa cells [5][6] | [IGFBP5](https://www.genecards.org/cgi-bin/carddisp.pl?gene=IGFBP5&keywords=IGFBP5) |
| **7** | **IGF1R** | Receptor tyrosine kinase which mediates IGF1 actions. It is required for FSH-mediated activation of the PI3K/AKT pro-survival cascade regulating follicle development. It is mainly localized in granulosa and theca cells [7] | [IGF1R](https://www.genecards.org/cgi-bin/carddisp.pl?gene=IGF1R&keywords=IGF1R) |
| **8** | **RHEB** | Small G-protein involved in follicular differentiation. Its activity is required for mTOR signaling, which is downstream of FSH in granulosa cells [8] | [RHEB](https://www.genecards.org/cgi-bin/carddisp.pl?gene=RHEB&keywords=RHEB) |
| **9** | **RPS6KB1** | Serine/threonine-protein kinase downstream effector of mTOR. In oocytes is important in maintaining the survival of primordial and developing follicles [9][10] | [RPS6KB1](https://www.genecards.org/cgi-bin/carddisp.pl?gene=RPS6KB1&keywords=RPS6KB1) |
| **10** | **RPTOR** | Subunit of mTORC1. Mice oocytes ablation exhibits similar follicular development and female fertility as wild-type mice. It is involved in spindle assembly in cumulus cell mitosis and oocyte meiosis [11][12] | [RPTOR](https://www.genecards.org/cgi-bin/carddisp.pl?gene=RPTOR&keywords=RPTOR) |
| **11** | **TSC2** | Suppressor of mTORC1 activity. In oocyte it is a part of an inhibitory mechanisms that maintain quiescence of primordial follicles [13] | [TSC2](https://www.genecards.org/cgi-bin/carddisp.pl?gene=TSC2&keywords=TSC2) |
| **13** | **MLST8** | Subunit of mTORC1 complex. Its genetic depletion affects embryogenesis whereas its specific role in ovarian folliculogenesis has to be explored [14][15] | [MLST8](https://www.genecards.org/cgi-bin/carddisp.pl?gene=MLST8&keywords=MLST8) |
| **14** | **MAPKAP1** | Kinase subunit of mTORC2. Different levels were found in follicular fluids depending on follicular developmental stage in yaks [16] | [MAPKAP1](https://www.genecards.org/cgi-bin/carddisp.pl?gene=MAPKAP1&keywords=MAPKAP1) |
| **15** | **RICTOR** | Subunit of mTORC2 which regulates folliculogenesis, follicle survival and female fertility. Mice oocyte ablation leads to POF phenotype including massive follicular atresia. It also controls spindle migration during meiotic oocyte maturation [15] [17][12] | [RICTOR](https://www.genecards.org/cgi-bin/carddisp.pl?gene=RICTOR&keywords=RICTOR) |
| **16** | **EIF4EBP1** | Repressor of translation initiation protein that has a key role in meiotic spindle formation of oocyte. It is expressed in oocyte [18] | [EIF4EBP1](https://www.genecards.org/cgi-bin/carddisp.pl?gene=EIF4EBP1&keywords=EIF4EBP1) |
| **17** | **FOXO1** | Transcription factor with a critical role in promoting follicular atresia and granulosa cell apoptosis. It plays also a role in the regulation of granulosa cells cycle upon hypoxia stimulation. It mainly expressed in granulosa cells [19][20][21] | [FOXO1](https://www.genecards.org/cgi-bin/carddisp.pl?gene=FOXO1&keywords=FOXO1) |
| **18** | **FOXO3** | Transcriptional activator that recognizes and binds to DNA. It regulates primordial follicle recruitment and activation. It is mainly expressed in the oocyte [22][23][24] | [FOXO3](https://www.genecards.org/cgi-bin/carddisp.pl?gene=FOXO3&keywords=FOXO3) |
| **19** | **SIRT1** | Nuclear deacetylase mainly expressed in granulosa cells and oocyte. It has a role in the adaptive response to oxidative stress as well as in protecting oocyte against loss of developmental competence during aging. Null female mice appear not to cycle efficiently through oestrous and ovulation does not occur although follicles develop normally [25] | [SIRT1](https://www.genecards.org/cgi-bin/carddisp.pl?gene=SIRT1&keywords=SIRT1) |
| **20** | **VEGFB** | Endothelial cell-derived growth factor B which might have a role in promoting angiogenesis in ovulatory follicles. Its expression has been found to be increased progressively from primary to antral sheep follicles [26] | [VEGFB](https://www.genecards.org/cgi-bin/carddisp.pl?gene=VEGFB&keywords=VEGFB) |
| **21** | **VEGFC** | Endothelial cell-derived growth factor C which might have a role in promoting angiogenesis in ovulatory follicles as has been found enriched in follicular fluids of granulosa cell hCG conditioned media [27] | [VEGFC](https://www.genecards.org/cgi-bin/carddisp.pl?gene=VEGFC&keywords=VEGFC) |
| **22** | **PGF** | Growth factor which plays a key role in follicular angiogenesis, ovulation and luteinization. Mainly expressed in theca cells [28] | [PGF](https://www.genecards.org/cgi-bin/carddisp.pl?gene=PGF&keywords=PGF) |
| **23** | **FGF1** | Theca and granulosa cell-derived growth factor. mRNA expression modulation might be implicated in different functions in relation to angiogenesis during follicle maturation, ovulation and corpus luteum formation [29] | [FGF1](https://www.genecards.org/cgi-bin/carddisp.pl?gene=FGF1&keywords=FGF1) |
| **24** | **FGF9** | Growth factor with a paracrine role in regulating ovarian progesterone production. It is also implicated in the regulation of granulosa and theca cell proliferation and cell cycle proteins. It is mainly localized in theca, stromal cells, and corpus luteum [30][31] | [FGF9](https://www.genecards.org/cgi-bin/carddisp.pl?gene=FGF9&keywords=FGF9) |
| **25** | **NOS3** | Reactive free radical acts as a biologic mediator in follicular and luteal angiogenesis. It is mainly localized in theca cells [32] | [NOS3](https://www.genecards.org/cgi-bin/carddisp.pl?gene=NOS3&keywords=NOS3) |
| **26** | **KDR** | Tyrosine-protein kinase receptor 2 for VEGFA, VEGFC, VEGFD playing an essential role in follicular angiogenesis. It is expressed in granulosa and thecal cells [33] | [KDR](https://www.genecards.org/cgi-bin/carddisp.pl?gene=KDR&keywords=KDR) |
| **27** | **NRP1** | Tyrosine kinase coreceptor which enhances follicle assembly. When upregulated in cumulus cells it may indicate an oocyte with a positive pregnancy outcome. Its expression in granulosa and somatic cells senses sex steroids [34][35] | [NRP1](https://www.genecards.org/cgi-bin/carddisp.pl?gene=NRP1&keywords=NRP1) |
| **28** | **FGFR1** | Growth factor receptor mainly expressed in cumulus cells and oocyte. mRNA expression is stimulated by FSH and might have a role in the oocyte maturation and cumulus activity around ovulation. It is modulated during the final stages of oocyte maturation [36][37] | [FGFR1](https://www.genecards.org/cgi-bin/carddisp.pl?gene=FGFR1&keywords=FGFR1) |
| **29** | **FLT1** | Tyrosine-protein kinase receptor 1 for VEGFA, VEGFB and PGF playing an essential role in follicular angiogenesis. It is expressed in granulosa and thecal cells [33] | [FLT1](https://www.genecards.org/cgi-bin/carddisp.pl?gene=FLT1&keywords=FLT1) |
| **30** | **HSP90AA1** | Ubiquitous heat shock protein whose expression is influenced by stressors during the follicular phase. It is also involved in the meiotic maturation of the oocyte [38][39] | [HSP90AA1](https://www.genecards.org/cgi-bin/carddisp.pl?gene=HSP90AA1&keywords=HSP90AA1) |

**Supplementary File 10. List of predicted interactor molecules.** For each gene the main biological function in the ovarian context has been reported. The table also includes the link to the Gene cards database (<https://www.genecards.org>) providing comprehensive information on all annotated and predicted genes. The knowledgebase automatically integrates gene-centric data from ~150 web sources, including genomic, transcriptomic, proteomic, genetic, clinical and functional information. Growth factors and intracellular signalling effectors involved in the metabolic control (grey scale color), and in the angiogenetic processes (green color) of the *iv*F network.

**References:**

1. Bulun SE, Wan Y, Matei D (2019) Epithelial mutations in endometriosis: Link to ovarian cancer. Endocrinology 160:626–638. https://doi.org/10.1210/en.2018-00794

2. Ramalingam P (2016) Morphologic, Immunophenotypic, and Molecular Features of Epithelial Ovarian Cancer. Oncology (Williston Park).

3. Richani D, Gilchrist RB (2018) The epidermal growth factor network: Role in oocyte growth, maturation and developmental competence. Hum Reprod Update. https://doi.org/10.1093/humupd/dmx029

4. Law NC, Hunzicker-Dunn ME (2016) Insulin receptor substrate 1, the hub linking follicle-stimulating hormone to phosphatidylinositol 3-kinase activation. J Biol Chem. https://doi.org/10.1074/jbc.M115.698761

5. Mazerbourg S, Monget P (2018) Insulin-like growth factor binding proteins and IGFBP proteases: A dynamic system regulating the ovarian folliculogenesis. Front. Endocrinol. (Lausanne).

6. Bøtkjær JA, Pors SE, Petersen TS, et al (2019) Transcription profile of the insulin-like growth factor signaling pathway during human ovarian follicular development. J Assist Reprod Genet. https://doi.org/10.1007/s10815-019-01432-x

7. Ipsa E, Cruzat VF, Kagize JN, et al (2019) Growth Hormone and Insulin-Like Growth Factor Action in Reproductive Tissues. Front. Endocrinol. (Lausanne).

8. Alam H, Maizels ET, Park Y, et al (2004) Follicle-stimulating Hormone Activation of Hypoxia-inducible Factor-1 by the Phosphatidylinositol 3-Kinase/AKT/Ras Homolog Enriched in Brain (Rheb)/Mammalian Target of Rapamycin (mTOR) Pathway Is Necessary for Induction of Select Protein Markers of Follic. J Biol Chem 279:19431–19440. https://doi.org/10.1074/jbc.M401235200

9. Reddy P, Adhikari D, Zheng W, et al (2009) PDK1 signaling in oocytes controls reproductive aging and lifespan by manipulating the survival of primordial follicles. Hum Mol Genet. https://doi.org/10.1093/hmg/ddp217

10. Sobinoff AP, Sutherland JM, Mclaughlin EA (2013) Intracellular signalling during female gametogenesis. Mol. Hum. Reprod.

11. Gorre N, Adhikari D, Lindkvist R, et al (2014) mTORC1 signaling in oocytes is dispensable for the survival of primordial follicles and for female fertility. PLoS One. https://doi.org/10.1371/journal.pone.0110491

12. Kogasaka Y, Hoshino Y, Hiradate Y, et al (2013) Distribution and association of mTOR with its cofactors, raptor and rictor, in cumulus cells and oocytes during meiotic maturation in mice. Mol Reprod Dev. https://doi.org/10.1002/mrd.22166

13. Adhikari D, Flohr G, Gorre N, et al (2009) Disruption of Tsc2 in oocytes leads to overactivation of the entire pool of primordial follicles. Mol Hum Reprod. https://doi.org/10.1093/molehr/gap092

14. Guertin DA, Stevens DM, Thoreen CC, et al (2006) Ablation in Mice of the mTORC Components raptor, rictor, or mLST8 Reveals that mTORC2 Is Required for Signaling to Akt-FOXO and PKCα, but Not S6K1. Dev Cell. https://doi.org/10.1016/j.devcel.2006.10.007

15. Guo Z, Yu Q (2019) Role of mTOR Signaling in Female Reproduction. Front. Endocrinol. (Lausanne).

16. Tao J, Zhao G, Zhao X, et al (2014) Proteomic analysis of the follicular fluid of Tianzhu white yak during diestrus. Int J Mol Sci. https://doi.org/10.3390/ijms15034481

17. Chen Z, Kang X, Wang L, et al (2015) Rictor/mTORC2 pathway in oocytes regulates folliculogenesis, and its inactivation causes premature ovarian failure. J Biol Chem. https://doi.org/10.1074/jbc.M114.605261

18. Severance AL, Latham KE (2018) Meeting the meiotic challenge: Specializations in mammalian oocyte spindle formation. Mol. Reprod. Dev.

19. Shen M, Liu Z, Teng Y, et al (2014) Involvement of FoxO1 in the effects of folliclestimulating hormone on inhibition of apoptosis in mouse granulosa cells. Cell Death Dis 5:1–14. https://doi.org/10.1038/cddis.2014.400

20. Li C, Liu Z, Zhou J, et al (2020) Insulin-like growth factor-I prevents hypoxia-inducible factor-1 alpha-dependent G1/S arrest by activating cyclin E/cyclin-dependent kinase2 via the phoshatidylinositol-3 kinase/AKT/forkhead box O1/Cdkn1b pathway in porcine granulosa cells. Biol Reprod. https://doi.org/10.1093/biolre/ioz162

21. Zielak-Steciwko AE, Evans ACO (2016) Genomic portrait of ovarian follicle growth regulation in cattle. Reprod Biol. https://doi.org/10.1016/j.repbio.2016.07.003

22. Castrillon DH, Miao L, Kollipara R, et al (2003) Suppression of ovarian follicle activation in mice by the transcription factor Foxo3a. Science (80- ). https://doi.org/10.1126/science.1086336

23. Shah JS, Sabouni R, Cayton Vaught KC, et al (2018) Biomechanics and mechanical signaling in the ovary: a systematic review. J. Assist. Reprod. Genet.

24. Lee HN, Chang EM (2019) Primordial follicle activation as new treatment for primary ovarian insufficiency. Clin. Exp. Reprod. Med.

25. Tatone C, Di Emidio G, Vitti M, et al (2015) Sirtuin Functions in Female Fertility: Possible Role in Oxidative Stress and Aging. Oxid. Med. Cell. Longev.

26. Ortega HH, Veiga-Lopez A, Sreedharan S, et al (2015) Developmental programming: Does prenatal steroid excess disrupt the ovarian VEGF system in sheep? Biol Reprod. https://doi.org/10.1095/biolreprod.115.131607

27. Kim SO, Trau HA, Duffy DM (2017) Vascular endothelial growth factors C and D may promote angiogenesis in the primate ovulatory follicle. Biol Reprod. https://doi.org/10.1095/biolreprod.116.144733

28. Bender HR, Trau HA, Duffy DM (2018) Placental growth factor is required for ovulation, luteinization, and angiogenesis in primate ovulatory follicles. Endocrinology. https://doi.org/10.1210/en.2017-00739

29. Berisha B, Welter H, Shimizu T, et al (2006) Expression of fibroblast growth factor 1 (FGF1) and FGF7 in mature follicles during the periovulatory period after GnRH in the cow. J Reprod Dev. https://doi.org/10.1262/jrd.17077

30. Drummond AE, Tellbach M, Dyson M, Findlay JK (2007) Fibroblast growth factor-9, a local regulator of ovarian function. Endocrinology. https://doi.org/10.1210/en.2006-1668

31. Totty ML, Morrell BC, Spicer LJ (2017) Fibroblast growth factor 9 (FGF9) regulation of cyclin D1 and cyclin-dependent kinase-4 in ovarian granulosa and theca cells of cattle. Mol Cell Endocrinol. https://doi.org/10.1016/j.mce.2016.11.002

32. Tessaro I, Luciano AM, Franciosi F, et al (2011) The endothelial nitric oxide synthase/nitric oxide system is involved in the defective quality of bovine oocytes from low mid-antral follicle count ovaries. J Anim Sci. https://doi.org/10.2527/jas.2010-3714

33. Araújo VR, Duarte ABG, Bruno JB, et al (2013) Importance of vascular endothelial growth factor (VEGF) in ovarian physiology of mammals. Zygote. https://doi.org/10.1017/S0967199411000578

34. Assidi M, Montag M, Van Der Ven K, Sirard MA (2011) Biomarkers of human oocyte developmental competence expressed in cumulus cells before ICSI: A preliminary study. J Assist Reprod Genet. https://doi.org/10.1007/s10815-010-9491-7

35. Shimizu T, Jayawardana BC, Nishimoto H, et al (2006) Hormonal regulation and differential expression of neuropilin (NRP)-1 and NRP-2 genes in bovine granulosa cells. Reproduction. https://doi.org/10.1530/rep.1.00937

36. Zhang K, Ealy AD (2012) Disruption of fibroblast growth factor receptor signaling in bovine cumulus-oocyte complexes during in vitro maturation reduces subsequent embryonic development. Domest Anim Endocrinol. https://doi.org/10.1016/j.domaniend.2011.12.006

37. Price CA (2016) Mechanisms of fibroblast growth factor signaling in the ovarian follicle. J. Endocrinol.

38. Abdelnour SA, Swelum AA, Abd El-Hack ME, et al (2020) Cellular and functional adaptation to thermal stress in ovarian granulosa cells in mammals. J. Therm. Biol.

39. Liu YH, Liu XM, Wang PC, et al (2018) Heat shock protein 90α couples with the MAPK-signaling pathway to determine meiotic maturation of porcine oocytes. J Anim Sci. https://doi.org/10.1093/jas/sky213
