# Supplementary material for: In Vitro Folliculogenesis in Mammalian Models: A Computational Biology Study
Source: Front Mol Biosci. 2021 Nov 9;8:737912. doi: 10.3389/fmolb.2021.737912 (PMC8630647; doi:10.3389/fmolb.2021.737912)
Supplement: Supplementary file 1 [file DataSheet1.ZIP › SUPPL FILES Frontiers Mol Bio/Suppl File 2.docx]

Supplementary Material

Supplementary File 2

| **Parameter** | **Definition** |
| --- | --- |
| Connected Components | It is the number of networks in which any two vertices are connected to each other by links, and which is connected to no additional vertices in the network. |
| Number of nodes | It is the total number of molecules involved. |
| Number of edges | It is the total number of interactions found. |
| Clustering coefficient | It is calculated as *C*I = 2*n*I/*k*I(*k*I–1), where *n*I is the number of links connecting the *k*I neighbours of node I to each other. It is a measure of how the nodes tend to form clusters. |
| Network diameter | It is the longest of all the calculated shortest paths in a network. |
| Shortest paths | The length of the shortest path between two nodes *n* and *m* is *L*(*n*,*m*). The **s**hortest path length distribution gives the number of node pairs (*n*,*m*) with *L*(*n*,*m*) = *k* for *k = 1,2,…* |
| Characteristic path length | It is the expected distance between two connected nodes. |
| Averaged number of neighbours | It is the mean number of connections of each node. |
| Node degree | It is the number of interactions of each node. |
| Node degree distribution | It represents the probability that a selected node has *k* links. |
| γ | Exponent of node degree equation. |
| R^2^ | Coefficient of determination of node degree vs. number of nodes, on logarithmized data. |

**Suppl. File 2.** Main topological parameters assessed in this study
