# Supplementary material for: In Vitro Folliculogenesis in Mammalian Models: A Computational Biology Study
Source: Front Mol Biosci. 2021 Nov 9;8:737912. doi: 10.3389/fmolb.2021.737912 (PMC8630647; doi:10.3389/fmolb.2021.737912)
Supplement: Supplementary file 1 [file DataSheet1.ZIP › SUPPL FILES Frontiers Mol Bio/Suppl File 4.docx]

Supplementary Material

Supplementary File 4

**a.**

| **Rank** | **Name** | **Link Count** | **Clustering Coefficient** |
| --- | --- | --- | --- |
| **1** | **FSH** | **155** | 0,0252809 |
| **2** | **PA*** FOLLICLE GROWTH** | **139** | 0,01772152 |
| **3** | **PRIM* TO P** FOL# TRANSITION** | **135** | 0,02054795 |
| **4** | **MEIOTIC COMPETENCE** | **112** | 0,00770308 |
| **5** | **ANTRUM DIFFERENTIATION** | **93** | 0,02175141 |
| **6** | **activin A** | **84** | 0,06089744 |
| **7** | **LH** | **65** | 0,01935874 |
| **8** | **IGF1** | **63** | 0,04423077 |
| **9** | **GDF9** | **60** | 0,05128205 |
| **10** | **E2** | **58** | 0,03429952 |
| **11** | **insulin** | **58** | 0,04048583 |
| **12** | **KL** | **55** | 0,07846154 |
| **13** | **EGF** | **54** | 0,04634581 |
| **14** | **P4** | **46** | 0,02820513 |
| **15** | **APOPTOSIS** | **40** | 0,01801802 |
| **16** | **bFGF** | **40** | 0,05263158 |
| **17** | **AMH** | **37** | 0,05632184 |
| **18** | **CELL SURVIVAL** | **37** | 0,03846154 |
| **19** | **CELL PROLIFERATION** | **37** | 0,03565062 |
| **20** | **PI3K** | **35** | 0,05434783 |
| **21** | **AKT** | **33** | 0,04093567 |
| **22** | **CYP19A1** | **33** | 0,04365079 |
| **23** | **VEGFA** | **32** | 0,03947368 |
| **24** | **PRIM* FOL^#^ ACTIVATION** | **31** | 0,03276353 |
| **25** | **hCG** | **30** | 0,00615385 |
| **26** | **FSHR** | **30** | 0,07312253 |
| **27** | **PA*** TO A^§^ FOL^#^ TRANSITION** | **30** | 0,06432749 |
| **28** | **PI3K + AKT** | **30** | 0,03333333 |
| **29** | **STEROIDOGENESIS** | **29** | 0,05230769 |
| **30** | **CELL DIFFERENTIATION** | **27** | 0,06666667 |
| **31** | **TGFbeta** | **26** | 0,05952381 |
| **32** | **cAMP** | **26** | 0,06190476 |
| **33** | **BMP15** | **25** | 0,06666667 |
| **34** | **LIF** | **24** | 0,05238095 |
| **35** | **insulin + FSH** | **24** | 0,17857143 |
| **36** | **OOCYTE GROWTH** | **23** | 0,02164502 |
| **37** | **BMP4** | **23** | 0,06190476 |
| **38** | **FST** | **23** | 0,08455882 |

**b.**

| **Rank** | **Name** | **BN Score** |
| --- | --- | --- |
| **1** | **MEIOTIC COMPETENCE** | 132.0 |
| **2** | **PRIM* TO P** FOL# TRANSITION** | 71.0 |
| **3** | **PA*** FOLLICLE GROWTH** | 56.0 |
| **4** | **FSH** | 55.0 |
| **5** | **EGF** | 38.0 |
| **6** | **LH** | 37.0 |
| **7** | **ANTRUM DIFFERENTIATION** | 31.0 |
| **7** | **insulin** | 31.0 |
| **9** | **activin A** | 28.0 |
| **10** | **P4** | 21.0 |
| **11** | **GDF9** | 20.0 |
| **12** | **APOPTOSIS** | 19.0 |
| **13** | **hCG** | 18.0 |
| **13** | **E2** | 18.0 |
| **13** | **cAMP** | 18.0 |
| **16** | **PRIM* FOL^#^ ACTIVATION** | 16.0 |
| **16** | **TGFbeta** | 16.0 |
| **16** | **CYP19A1** | 16.0 |
| **16** | **LIF** | 16.0 |
| **20** | **IGF1** | 14.0 |
| **21** | **BMP15** | 13.0 |
| **21** | **bFGF** | 13.0 |
| **23** | **STEROIDOGENESIS** | 12.0 |
| **23** | **CELL SURVIVAL** | 12.0 |
| **23** | **cumulin** | 12.0 |
| **26** | **AMH** | 11.0 |
| **26** | **mTOR** | 11.0 |
| **26** | **DHT** | 11.0 |
| **29** | **AKT** | 10.0 |
| **30** | **VEGF-A** | 9.0 |
| **30** | **Ca2+** | 9.0 |
| **32** | **SMAD4** | 8.0 |
| **32** | **SOMATIC CELL TO OOCYTE COMMUNICATION** | 8.0 |
| **32** | **FST** | 8.0 |
| **32** | **PA*** TO A^§^ FOL^#^ TRANSITION** | 8.0 |
| **32** | **SOCS4 + JAK + STAT** | 8.0 |
| **32** | **ATRESIA** | 8.0 |
| **32** | **CELL PROLIFERATION** | 8.0 |

**Suppl. File 4**

1. **Analysis of network hubs.** Edge count (link count) and clustering coefficient were computed to classify the hubs of the biological network made from WoS_MMi*v*F database (PRIM* primordial, P** Primary, PA*** preantral, A^§^ antral, and Fol^#^ Follicle).
2. **List of Bottleneck nodes (BN).** The top 38 BN were ranked on the basis of relative bottleneck score (PRIM* primordial, P** Primary, PA*** preantral, A§ antral and, Fol# Follicle).
