# Supplementary material for: In Vitro Folliculogenesis in Mammalian Models: A Computational Biology Study
Source: Front Mol Biosci. 2021 Nov 9;8:737912. doi: 10.3389/fmolb.2021.737912 (PMC8630647; doi:10.3389/fmolb.2021.737912)
Supplement: Supplementary file 1 [file DataSheet1.ZIP › SUPPL FILES Frontiers Mol Bio/Suppl File 5.docx]

Supplementary Material

Supplementary File 5


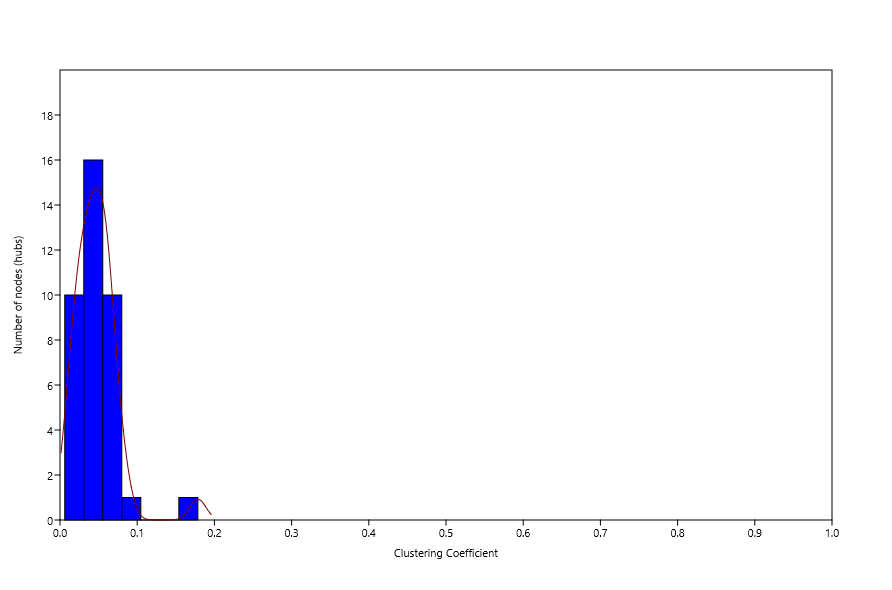


**Suppl File 5. KDE analysis.** Graph showing the KDE of the clustering coefficient of the hubs
